# Excitatory synchronization of rat hippocampal interneurons during network activation *in vitro*

**DOI:** 10.3389/fncel.2023.1129991

**Published:** 2023-03-09

**Authors:** Viktoria S. Pendeliuk, Igor V. Melnick

**Affiliations:** ^1^Hospital of Urgent Medical Care, Department of Surgery No. 4, NAMS of Ukraine, Kiev, Ukraine; ^2^Department of Biophysics of Ion Channels, Bogomoletz Institute of Physiology, NAS of Ukraine, Kiev, Ukraine

**Keywords:** synaptic transmission, GABA, glutamate, hippocampus, interneurons, synchronization, gap junctions

## Abstract

**Introduction:**

Hippocampal interneurons (INs) are known to synchronize their electrical activity *via* mechanisms, which are poorly defined due to immense complexity of neural tissue but seem to depend on local cell interactions and intensity of network activity.

**Methods:**

Here, synchronization of INs was studied using paired patch-clamp recordings in a simplified culture model with intact glutamate transmission. The level of network activity was moderately elevated by field electric stimulation, which is probably an analogue of afferent processing *in situ*.

**Results:**

Even in baseline conditions, ∼45% of spontaneous inhibitory postsynaptic currents (sIPSCs) resulting from firing of individual presynaptic INs coincided between cells within ±1 ms due to simple divergence of inhibitory axons. Brief network activation induced an appearance of ‘hypersynchronous’ (∼80%) population sIPSCs occurring in response to coherent discharges of several INs with jitter ±4 ms. Notably, population sIPSCs were preceded by transient inward currents (TICs). Those were excitatory events capable to synchronize firing of INs, in this respect being reminiscent of so-called fast prepotentials observed in studies on pyramidal neurons. TICs also had network properties consisting of heterogeneous components: glutamate currents, local axonal and dendritic spikelets, and coupling electrotonic currents likely *via* gap junctions; putative excitatory action of synaptic gamma-aminobutyric acid (GABA) was not involved. The appearance of population excitatory-inhibitory sequences could be initiated and reproduced by firing of a single excitatory cell reciprocally connected with one IN.

**Discussion:**

Our data demonstrate that synchronization of INs is initiated and dominated by glutamatergic mechanisms, which recruit, in a whole-sale manner, into supporting action other excitatory means existing in a given neural system.

## 1. Introduction

In higher brain structures as the neocortex and hippocampus, the cells within a local group or even widely separated tend to display electrical activity synchronous in the second and millisecond time scale, which is important for physiological states such as sensory processing, sleep or arousal, and in pathological conditions as epilepsy (Engel et al., 1992; Yuste et al., 1992; Harris and Gordon, 2015). This synchrony remains one of the most enigmatic properties of the brain, the mechanisms of which have been studied for decades. It is thought that four general processes could underlie such coherence: excitatory synapses releasing glutamate (Glu), electrotonic coupling *via* gap junctions, electrical field effects (ephaptic interactions), and changes of extracellular ions (Dudek et al., 1986); the expression of which seem to depend on the cell type, brain region, animal species, developmental age, and experimental conditions. While this classical knowledge has been mainly obtained in studies of excitatory pyramidal cells, much less is known for inhibitory interneurons (INs).

Interneurons represent a highly variable and distinct population of nerve cells in cortical structures depending on the layer location, dendritic pattern, axonal projections, and neuropeptide phenotype (Freund and Buzsaki, 1996). The subsets of INs innervate both pyramidal cells and each other in a specific manner releasing gamma-aminobutyric acid (GABA) (Pfeffer et al., 2013; Karnani et al., 2016). The latter binds to GABA_*A*_ receptors opening Cl^–^ channels, which depresses postsynaptic electrogenesis *via* both membrane hyperpolarization and shunting effects (Miles et al., 1996; Vida et al., 2006). Moreover, INs are thought to operate like a network and synchronize their own activity *via* mechanisms to some extent distinct from those of pyramidal cells. Accordingly, to current knowledge, two specific processes might mediate this synchrony endogenously and independently of Glu neurotransmission. First, pioneering studies of Ben-Ari et al. (1989) discovered that neonatal hippocampal neurons display synchronous bursting episodes mediated by GABA_*A*_ receptors, so-called giant-depolarizing potentials (GDPs). These authors have proposed that, in contrast to adult cells, GABA has an unusual depolarizing and excitatory action in connections between INs due to elevated intracellular Cl^–^ at that immature developmental stage (Khazipov et al., 1997; Dzhala and Staley, 2003; Sipilä et al., 2005). On the other hand, this mechanism has been also found in mature animals, in a fraction of INs expressing neuropeptide Y (Fu and van den Pol, 2007), in axo-axonic cells (Szabadics et al., 2006), and during epileptiform activity induced by convulsant drugs or tetanic electrical stimulation (Avoli et al., 1993; Michelson and Wong, 1994; Benardo, 1997; Bracci et al., 1999; Velazquez and Carlen, 1999). Second, morphological studies have found or suggested the presence of gap junctions in the dendrites of INs (Gulyás et al., 1996; Fukuda and Kosaka, 2000; Shigematsu et al., 2019), which would allow a direct transfer of electrical currents between cells facilitating the firing of action potentials (APs) and their coherence. The impact of gap junctional coupling on synchrony has been extensively studied mainly in neocortical neurons leading to high-frequency coherent oscillations of extracellular field and intracellular membrane potentials, which are observed during a range of behaviors *in vivo* or induced *in vitro* either chemically or by electrical stimulation (Steriade et al., 1993; Zhang et al., 1998; Skinner et al., 1999; Beierlein et al., 2000; Bartos et al., 2002).

The classical body of evidence indicates that excitation of individual INs is provided from external sources, in a feed-forward manner from long-ranged afferent fibers and in a feed-back (recurrent) way from local excitatory cells, both releasing Glu acting on ionotropic and metabotropic receptors, iGluR and mGluR, respectively (Miles, 1990a; McBain and Dingledine, 1993; Miles and Poncer, 1993; Geiger et al., 1997; Frerking et al., 1998; Semyanov and Kullmann, 2001; Kerlin et al., 2010; Karnani et al., 2016). It is not very surprising that the synchrony of INs can be simply induced by their near-simultaneous activation by coherent sensory input, which was demonstrated as intracellular Ca^2+^ waves synchronous within chemically-defined subsets of cortical cells (Karnani et al., 2016). Apart from this, the role of traditional Glu-ergic processes in synchronizing the activity of INs has been rarely addressed directly so far. It has been primarily due to the immense complexity in the synaptic organization of neural tissue and thus, of its electrical epiphenomena (e.g., Shigematsu et al., 2019). In particular, it is still not known with certainty how are generated those network electrical events, i.e., GDPs, epileptiform bursts, and electrical oscillations, which currently serve as models of neural synchronization. They reflect the patterned activity of thousands of nerve cells and it is difficult to isolate the activity of individual INs without interfering with excitatory cells and using respective antagonists. Thus, possibly one of the main controversies still centers around the nature of INs excitation, whether it is endo- or exogenous (i.e., dependent on iGluR). In the example of GDPs initially thought as purely GABAergic, the excitatory Glu currents within depolarizing episodes were found more recently and GDPs revealed sensitivity to blockers of iGluR (Khazipov et al., 1997; Khalilov et al., 1999). Induced epileptiform discharges were reported to persist under the blockers, when the network was made hyperexcitable (Michelson and Wong, 1994), while the blockers did affect some components of interictal- and ictal-like activity in other studies (Avoli et al., 1993; Bracci et al., 1999; Velazquez and Carlen, 1999). As for the oscillations, they were abolished by antagonists of iGluR in the study of Fisahn et al. (1998) but were resistant to them in experiments of Whittington et al. (1995). Still another limitation of existing models is that they correspond to an already high level of network activity, which could affect cellular synchrony; at the same time, the initial state often remains unknown.

In attempts to elucidate intrinsic and basic mechanisms of INs synchronization, here we present a novel and simplified approach by using low-density culture and assuming that synchrony in the hippocampus is of local character resulting from interactions of a few neighboring cells, the idea expressed by several authors (Ben-Ari et al., 1989; Khazipov et al., 1997). The correlation of spontaneous inhibitory postsynaptic currents (sIPSCs) in cell pairs was studied in the course of moderate network activation and with intact iGluR, which presumably is analogous to natural afferent processing. We demonstrate the appearance of population sIPSCs resulting from the firing of a few presynaptic INs. Population sIPSCs were preceded by transient inward currents (TICs) serving as excitatory and synchronizing network events of different natures. They consisted of dominant Glu-mediated components, coupling electrotonic currents (presumably, *via* gap junctions) and local dendritic and axonal spikelets. The latter, in its turn, represented a novel mechanism contributing to the local synchrony of synaptic inhibition.

## 2. Materials and methods

All animal procedures here conformed to the principles of worldwide regulations (Grundy, 2015). The experiments were carried out according to guidelines approved according to Protocol no. 3/14 from 06.2015 from the Bogomoletz Institute of Physiology (Ukraine) and as regulated by the European Community Council Directive (2010/63/EU).

### 2.1. Hippocampal culture

Newborn rats (0–1 day) were anesthetized with instant decapitation. Hippocampi were dissociated enzymatically with 0.05% pronase E (Serva) and gentle trituration. Cells were plated at a density of 4–5 × 10^4^ cells cm^–2^ on glass coverslips coated with a mixture of laminin/poly-L-ornithine. The feeding medium consisted of minimal essential medium (MEM), 0.6% glucose, 1 mM glutamine, 26 mM NaHCO_3_, 0.01 mg/ml insulin, 0.1 mg/ml holo-transferrin (Sigma), and 10% horse serum (Gibco). The medium was changed 1–2 times per week. The cultures were kept at 37°C in humidified air with 5% CO_2_ and after 2 weeks *in vitro* were used for experiments.

### 2.2. Electrophysiology

**FIGURE 1:**
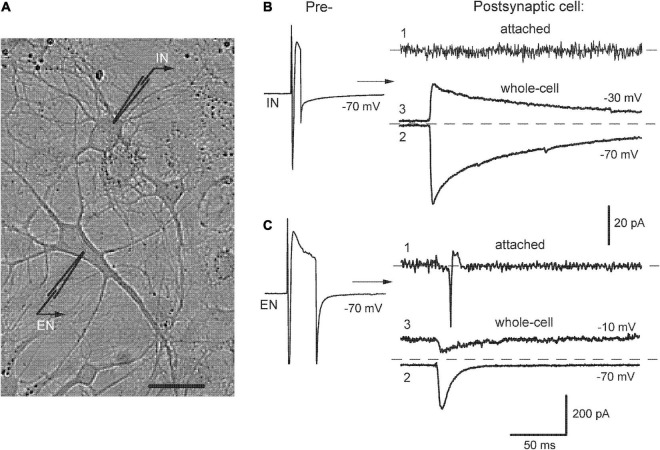
Identification of neurons in pair recordings. **(A)** A photograph of mutually connected inhibitory-excitatory neurons (IN-EN pair) in culture, bar 20 μm. **(B,C)** *Left*, stimulation of presynaptic cells to evoke inward Na^+^ currents. *Right*, respective postsynaptic responses recorded consecutively in cell-attached (1) and whole-cell mode at indicated membrane potentials (2, 3). From here on, such values are designated near the traces.

**FIGURE 2:**
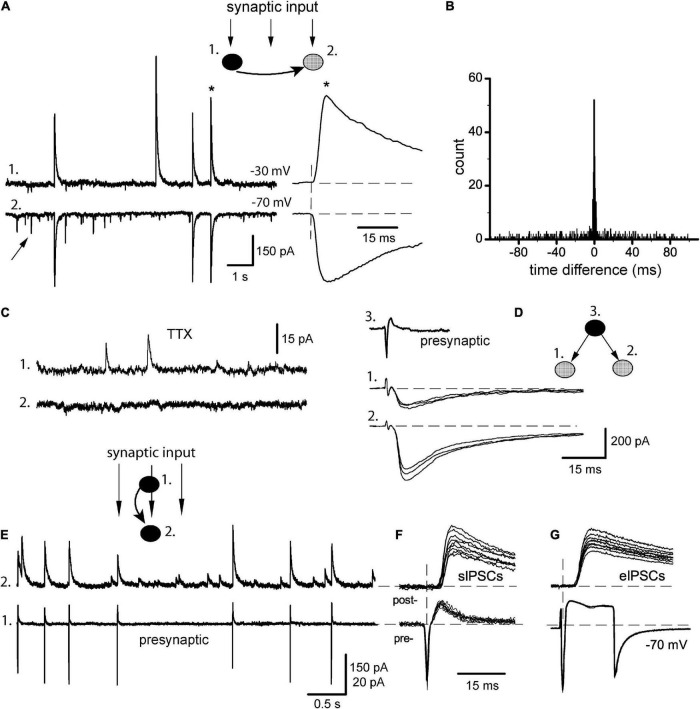
Synchrony of baseline sIPSCs. **(A)** Classical pair recording of incoming synaptic input in a pair of IN-unidentified cells (*1* and *2*, respectively), from here and below the phenotype of each pair is shown on *insets* (filled circles: INs, gray: unidentified cells). *Arrow* points on sEPSC; one of the coupled sIPSCs (*) is expanded to the right. **(B)** Respective time difference histogram. **(C)** Application of 0.5 μM TTX abolished synchrony of sIPSCs (same pair). **(D)** Stimulation of one presynaptic IN (*3*) evoked IPSCs in sequentially recorded neurons (*1*, *2*) showing the divergence of axons. **(E)** Identification of synaptic output of a given presynaptic neuron (*#1*, cell-attached mode) within a raw synaptic input impinging on postsynaptic neuron (*#2*, whole-cell mode). **(F)** Selection of APs-coupled sIPSCs. **(G)** Presynaptic stimulation with both neurons kept in whole-cell mode and recording unitary eIPSCs.

Patch-clamp recordings were made in cell-attached and whole-cell modes from pairs of neurons using two EPC-7 amplifiers (List, Germany). The extracellular solution (ACSF) contained (in mM): NaCl 140, KCl 4, CaCl_2_ 2, MgCl_2_ 1, HEPES 10, glucose 10. The intracellular solution contained (in mM): K-gluconate or Cs-gluconate (for postsynaptic neuron) 118, CsCl 12, MgCl_2_ 4, Na_2_ATP 4, EGTA 10, HEPES 10; pH of all solutions was 7.3. Patch pipettes had resistance 3–5 MΩ after filling with the intracellular solution. The access resistance was <25 MΩ at the beginning of an experiment and the data were discarded if it increased by >20%, series resistance compensation was not used. Liquid junction potential was measured as + 11 mV and was not corrected. The holding potential (V_*m*_) in cell-attached mode was adjusted to zero holding current, in the whole-cell mode it was −70 mV for presynaptic cell and typically between −30 and −20 mV for postsynaptic neuron, i.e., positive to Cl^–^ reversal potential (theoretical *E*_*Cl*_ = −50 mV at 20°C). Cell identification in a pair was achieved by their sequential stimulation (50-mV depolarizing pulses, 5–40 ms in duration and 0.2 Hz frequency) and recording of either outward or inward postsynaptic responses (Figure 1). During initial characterization, the outward currents at V_*m*_ −30 mV (*B*, trace 3) were blocked by the application of GABA_*A*_ receptor antagonist 10 μM bicuculline and were considered GABAergic inhibitory postsynaptic currents (IPSCs). Inward responses retained their direction at potentials up to 0 mV (*C*, trace 2–3) and were abolished by combined application of 50 μM APV and 10 μM CNQX (APV/CNQX), blockers of N-methyl-D-aspartate (NMDA) and non-NMDA subtypes of iGluR; they were considered as Glu-ergic excitatory postsynaptic currents (EPSCs). Similarly, using this separation by holding potential allowed us to visualize simultaneously inward sEPSCs and outward sIPSCs during the recording of spontaneous synaptic activity (e.g., Figure 2A).

The activation of neuronal networks was achieved by field electrical stimulation delivered to the whole coverslip by bipolar tungsten electrodes with poles separated by 10 mm and fed by constant 5–15 V voltage pulses from an isolating stimulator unit (model A365, World Precision Instruments, Sarasota, FL, USA). Stimulation protocol consisted of 3–7 trains separated by 5-s intervals, each of the trains having ten 1-ms pulses at 10 Hz frequency. The voltage of pulses was set at the lowest end and steadily increased between stimulation sessions until the minimal effects observable by the eye were noticed; among those, changes in the appearance of sIPSCs and/or their frequency were used as criteria of a positive outcome. The recordings started at 1 min after stimuli termination and continued for another 20 min; in preliminary experiments, it proved to be sufficient to cover ongoing changes before the network activity restores its baseline level. In some experiments, local extracellular stimulation of individual cells was performed. Single electrical pulses were delivered locally *via* a double-barreled micropipette with resistance 1–2 MΩ after filling with bath solution. The precision of stimulation was achieved by fine adjustment of its intensity and pipette position in close proximity to the dendrites or soma of a cell; moving the stimulating pipette a few micrometers away abolished APs, those were verified in cell-attached recording (Figure 7E). Membrane currents were low-pass filtered at 3 kHz, and sampled at 3–5 kHz using a computer interface (ITC-16 board, List, Germany) and TIDA acquisition software (List, Germany). The experiments were performed at room temperature (20–22°C).

Tetrodotoxin (TTX) was purchased from Sigma, and other substances such as bicuculline methiodide (BMI), D,L-2-amino-5-phosphonovaleric acid (APV), and 6-cyano-7-nitroquinoxaline-2,3-dione (CNQX) were obtained from RBI, they were dissolved in ACSF before use. Recordings were done in static bath conditions, while drug applications were performed locally by using a multibarrel system. For this, inflow and outflow pipettes (internal diameter 50 and 80 μm, respectively) were positioned with a separation of ∼400 μM between them so that an area containing a limited number of neurons (usually, <5) was covered. The procedure was initiated by applying only ACSF without drugs to obtain a steady-state level in the amplitudes of IPSCs and EPSCs. Between recordings, the bath was briefly superfused with ACSF to replenish its level.

### 2.3. Data analysis

Individual sIPSCs were extracted from continuous records using an event detection program (ANDATRA, Boychuk Y., Kiev). Only stable paired recordings were considered as judged from the running averages of 30 events. The detection criteria were set as reported elsewhere (Otis and Mody, 1992). Briefly, sIPSCs were inspected visually and apparent spurious and multiple detections of large events were rejected, the events with an amplitude >5 pA for miniature IPSCs and >20 pA for sIPSCs were acquired. The sIPSCs were analyzed semi-automatically. The following parameters were calculated for individual events: rise time (10–90%), peak amplitudes, and decay time constant (mono- or bi-exponential fitting using the non-linear least square method). Synaptic delay of spontaneous and evoked IPSCs was measured from the peak of presynaptic APs in cell-attached mode and inward Na^+^ currents in whole-cell mode, to the onset of IPSCs (Figures 2F, G).

In the analysis of the correlation between sIPSCs in pair recordings, time difference histograms were plotted as described elsewhere (Vincent and Marty, 1993), with minor modifications. Briefly, one of the channels was set as a reference. For each reference sIPSCs, the event closest in time was found in the partner trace and the time difference between them was entered. Because of the usually low frequency of spontaneous activity and negligible by-chance coincidence, coupled IPSCs (*C*) were readily discriminated by visual inspection from single (uncoupled) events (*U*) (e.g., Figure 2A). The value of *C* was estimated as the integral of the main peak in the histogram and *U* as an area outside of the peak. The coupling ratio was then defined as *R* = C/(C + U) × 100%. Results are given as mean ± SEM. Student’s paired and unpaired *t*-test was used when appropriate. The probability level *P* < 0.05 was considered significant.

## 3. Results

### 3.1. Identification of neurons

The neurotransmitter phenotype of studied cells was determined in pair recordings by stimulating them sequentially and observing evoked postsynaptic responses. Figure 1 illustrates this procedure in the example of mutually connected inhibitory-excitatory neurons (*A*, designated as IN-EN). Presynaptic stimulation of IN (B, left) evoked IPSCs in EN (right) directed inwardly at −70 mV (trace 2) and outwardly at −30 mV (trace 3). These currents reversed at −48.1 ± 3.5 mV (*n* = 12), which was close to Cl^–^ equilibrium potential. In turn, stimulation of EN (C, left) elicited EPSCs in IN (right), the responses at −70 and −10 mV are shown (*2*, *3*). They changed their direction at −1.8 ± 2 mV (*n* = 10) indicating permeability for cations. The decay of eIPSCs was much slower than that of eEPSCs. At −30 mV, it was approximated by fast and slow components in ∼70% of cells (the reasons for this complexity of the decay phase were not studied) and the time constant of the former was 24 ± 2.7 ms (*n* = 12). The decay of eEPSCs was mainly monoexponential with tau 7.8 ± 1.2 ms (*n* = 10). This identification of synaptic currents based on their direction and decay kinetics was initially confirmed by respective antagonists and did not require their constant use in further recordings. Thus, we commonly held postsynaptic neurons at −30 or −20 mV to provide better resolution of simultaneously recorded outward IPSCs and inward EPSCs.

This identification protocol was preceded by the observation of postsynaptic responses first in cell-attached mode, which preserved intact the concentration of intracellular Cl^–^ (B-C, traces 1). This provided information on whether presynaptic firing could evoke APs in postsynaptic cells. Stimulation of EN reliably evoked postsynaptic spikes in EN-IN pairs as expected (C, *n* = 11/12) but none of the tested presynaptic IN-induced APs in postsynaptic cells, either in IN-EN pairs (B, *n* = 8) or in pure IN-IN pairs (*n* = 7). Similar results were also obtained in IN-IN pairs (*n* = 9/10) after network activation induced by field electrical pulses.

### 3.2. Baseline synchrony of spontaneous IPSCs

Neurons in culture display variable spontaneous activity ranging from random events to intensive bursting discharges; the latter is sometimes called either epileptiform or oscillatory behavior (McBain et al., 1989; Bacci et al., 1999). In our hands, the neurons tended to show rare, isolated events at the onset of experiments, while any electrical stimulations or even inadvertent mechanical disturbances could lead to more intensive and complex patterns. Thus, the former type was assumed as a normal baseline activity of a non-stimulated network. Appropriately selected cells were recorded in pairs (*n* = 15) with at least one GABAergic neuron (Figure 2A, the phenotype of each pair is indicated on *insets*). One neuron of a pair was kept at −30 mV to display outward sIPSCs (trace 1) and their partner events were easily identified in another cell even at −70 mV (trace 2). Both sIPSCs and sEPSCs (arrow) occurred randomly with low frequency (1.8 ± 0.4 and 2.4 ± 0.6 Hz, respectively) and did not interfere with each other. Similar to evoked responses, sIPSCs had a slow bi-exponential decay in the majority of cells with a tau of fast component 21.5 ± 1.8 ms (*n* = 15 cells), while sEPSCs decayed rapidly with tau 8.2 ± 1.5 ms (*n* = 15 cells). Even in baseline conditions, many sIPSCs occurred synchronously in two cells, and coupling ratio *R* was calculated as 45.3 ± 2.8% (range 30–61%), with almost instantaneous precision (Figure 2A, asterisk). This corresponded to a narrow peak in time difference histograms centered at 0 ms with >95% of events grouped within ± 1 ms (synchronization width) (Figure 2B). Such tight synchrony could be due to the firing of a common presynaptic IN with divergent axon collaterals supplying both recorded cells (Miles, 1990b; Vincent and Marty, 1993). Really, the application of 0.5 μM TTX abolished high-amplitude events and their correlation (*n* = 5, Figure 2C); only miniature IPSCs with a mean amplitude of ∼10 pA remained. This explanation was further confirmed in triple recordings (*n* = 4, Figure 2D), where stimulation of a single IN in cell-attached mode (trace 3) could evoke IPSCs in two sequentially recorded postsynaptic cells (traces 1–2). Such paired whole-cell recordings as above are a common method to study the synchrony of postsynaptic events, at the same time the real activity of presynaptic cells remains unknown. The latter can be alleviated by the approach of Vincent and Marty (1996), when presynaptic IN is found and kept in cell-attached mode, while postsynaptic neuron is recorded whole-cell (Figure 2E). As a result, sIPSCs corresponding to APs in a given presynaptic IN could be identified within a raw synaptic input. When APs-matched sIPSCs (cell 2) were aligned to respective presynaptic APs (cell 1), it gave a group of sIPSCs with small variations in their characteristics (Figure 2F). As a next step, the presynaptic neuron was ruptured and stimulated in whole-cell mode producing unitary eIPSCs (Figure 2G). Not very surprisingly, the properties of those sIPSCs and eIPSCs proved to be almost identical, as was compared in six pairs: synaptic delay 2.67 ± 0.06 vs. 2.71 ± 0.05 ms (*t* = 1.76, *P* > 0.1), rise time 2.52 ± 0.06 vs. 2.60 ± 0.04 ms (*t* = 2.07, *P* > 0.05), amplitude 156 ± 11 vs. 167 ± 14 pA (*t* = 2.14, *P* > 0.05). We checked also the possibility that the properties of eIPSCs could depend on the type of presynaptic intracellular solution based either on K^+^ or Cs^+^ (Vincent and Marty, 1996). In our experiments, however, using hippocampal (but not cerebellar) neurons, it was not the case. These data again support the conclusion that sIPSCs in baseline conditions were unitary events representing the synaptic output of individual INs.

### 3.3. Effects of network activation

**FIGURE 3:**
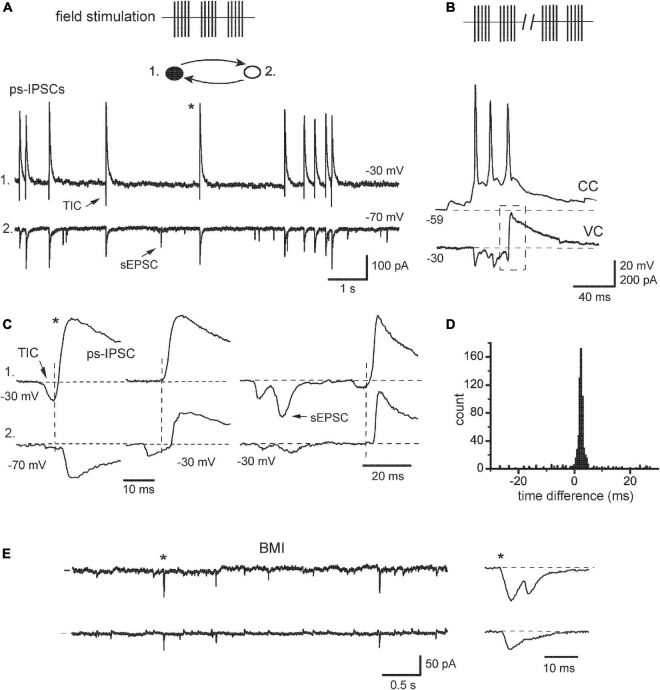
Transformation of sIPSCs after network activation. **(A)** “Hypersynchronous” sIPSCs observed after field electrical stimulation (*ps-IPSCs*), they were preceded by transient inward currents (*TIC*). *Inset*: filled circles, IN; open, EN. Random sEPSC is marked by an *arrow* [**(A,C–E)**, same recording]. **(B)** An example of epileptiform bursting episodes induced after prolonged stimulation. One cell in a pair was in current-clamp mode (*CC*) to show action potentials. The other cell was kept in a voltage-clamp (VC), which revealed an excitatory-inhibitory sequence (*dashed box*) as an intrinsic component of the bursting episode. **(C)** Different examples of sequences TIC/ps-IPSC are expanded to show the timing of the events (*vertical lines*), and *asterisk* shows the events taken from the segment in panel **(A)**. Cell #1 was constantly held at –30 mV, while cell #2 was held first at –70 and then at –30 mV. **(D)** Time difference histogram. **(E)** Application of bicuculline (BMI) blocked outward Cl^–^ currents and revealed synchronous excitatory currents; one of them is expanded to the *right*.

**FIGURE 4:**
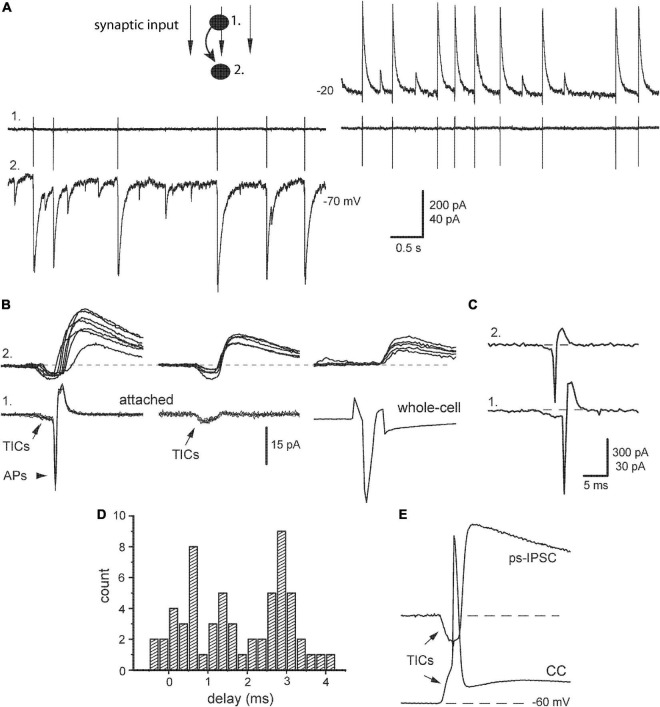
Relation of post-stimulus sIPSCs with presynaptic action potentials. **(A)** Identification of synaptic output of a given presynaptic neuron (#1, cell-attached mode) within a raw post-stimulus sIPSCs impinging on postsynaptic neuron (#2, whole-cell mode) at indicated potentials. **(B)** *Left*, paired segments were aligned, respectively to presynaptic action potentials (*APs*) with preceding TICs. *Middle*, examples of APs failures and persisting TICs. *Right*, presynaptic IN was stimulated next in whole-cell mode. **(C)** An example of correlated APs in pair of INs. **(D)** Distribution of synaptic delays [taken from panel **(A)**]. **(E)** An example of the paired segment with synchronous TICs when presynaptic IN was kept in current-clamp mode (CC).

Cultured neurons were briefly activated by field electrical stimulation and the effects on synchrony of sIPSCs were studied with intact synaptic transmission, i.e., without the presence of iGluR antagonists. Those have been commonly used so far in most experiments in slices to isolate inhibitory events, especially when extracellular stimulation was employed (Miles, 1990b; Miles and Poncer, 1993; Vincent and Marty, 1993; Michelson and Wong, 1994; Whittington et al., 1995; Hájos and Mody, 1997; Bartos et al., 2002). Weak stimulation induced a short-term (∼20 min) and moderate increase (<2 times) in the frequency of spontaneous events (Figure 3A, 17/25 pairs; see also Miles and Poncer, 1993). Stronger or more prolonged stimulation leads to more complex activities including epileptiform, which was seen in current-clamp mode as synchronous bursting depolarizations with APs (Figure 3B). We noticed intrinsic changes in post-stimulus sIPSCs (designated as ps-IPSCs) already after weak stimulation and focused on them (Figure 3A). The currents appeared similar to usual sIPSCs at −70 mV (Figure 3A, cell 2; Figure 4A, left) but depolarizing cells above *E*_*Cl*_ revealed that ps-IPSCs were preceded by transient inward currents (TICs) appearing as excitatory-inhibitory sequences, i.e., TICs/ps-IPSCs (Figure 3A, cell 1; Figure 4A, right). Random independent sEPSCs were also observed (Figures 3A, C, arrows). TICs had a small amplitude of 18 ± 8 pA at −30 mV (range 3–130 pA, *n* = 17) and variable shape presumably due to kinetically distinct components (Figure 3C). Some TICs could be seen even at −70 mV but mostly they were merged with the rising phase of ps-IPSCs. Interestingly, excitatory-inhibitory synaptic sequences could be also found as components within more complex bursting activity induced after stronger stimulation (Figure 3B, dashed box). Individual ps-IPSCs were highly correlated between cells and coupling ratio *R* increased to 81 ± 3.5% (range 64–95%; *n* = 17, *t* = 9.5, *P* < 0.001). In this case, time differences were measured using inflection points between TICs and ps-IPSCs (Figure 3C, dashed lines). Of note, ps-IPSCs in *cell 1* occurred earlier by ∼2.7 ms, a value comparable with synaptic delay. As a result, the main peak in the time difference histogram was shifted from 0 ms to the right (Figure 3D). Similar data were obtained in 11/17 pairs and six of those were of IN-EN type; in the other five pairs the histograms remained centered. Overall, the synchronization width was broader than in baseline sIPSCs and reached ± 4 ms. This type of elevated synchrony of ps-IPSCs characterized by the presence of excitatory pre-currents will be referred to below as excitatory. With a further purpose to isolate TICs and to determine how tight their link is with Cl^–^ currents, the application of GABA_*A*_ antagonist BMI 10 μM abolished IPSCs and revealed inward currents, which occurred independently and looked similar to sEPSCs (Figure 3E, 6/6 pairs). Those were often correlated between cells and therefore could be a source of elevated synchrony of ps-IPSCs.

To get a deeper insight into the nature of ps-IPSCs, we estimated their relations with spontaneous APs recorded from presynaptic neurons kept in cell-attached mode (Figure 4A, #1) and compared then selected ps-IPSCs with single-cell evoked IPSCs. Paired segments were aligned, respectively to APs revealing strong variation in corresponding ps-IPSCs (Figure 4B, left). It was clearly distinct from the behavior of evoked responses, those reflected a real contribution of a given presynaptic IN to ps-IPSCs and, notably, contained no excitatory pre-currents (right). Another obvious difference between ps-IPSCs and eIPSCs was in their synaptic delays, 1.23 ± 0.3 vs. 2.72 ± 0.05 ms (*n* = 7, *t* = 5.12, *P* < 0.01). The smaller mean delay of ps-IPSCs was due to their broad distribution starting even from negative values, from −0.4 to 4.2 ms in a given example (Figure 4D) and from −8 ms in one extreme case. Their rise time was slower than of eIPSCs, 3.73 ± 0.23 vs. 2.53 ± 0.06 ms (*n* = 7, *t* = 5.89, *P* < 0.01), and the amplitude larger, 259 ± 21 vs. 145 ± 7 pA (*n* = 7, *t* = 5.5, *P* < 0.01). Due to low noise in attached recordings, TICs could be frequently observed also in presynaptic IN along with APs (Figure 4B, arrows). This allowed us to find paired segments, where presynaptic APs failed in a given IN but ps-IPSCs of smaller amplitudes persisted (middle). These data directly show that ps-IPSCs were population (i.e., compound) events induced by the firing of >1 presynaptic INs, most likely 2–3. Those numbers were derived from frequent observations of correlated APs in both INs at the onset of cell-attached recordings (Figure 4C) and up to three peaks were discriminated in distributions of synaptic delays (Figure 4D).

Still another important conclusion could be drawn from these observations of TICs and following APs with failures in presynaptic cells, namely that TICs were causal events capable to evoke APs confirming their excitatory and synchronizing role. This idea was also illustrated further with presynaptic INs kept in current-clamp mode (Figure 4E). The latter is traditional for classical microelectrode recordings and provides a link to original studies, where similar electrical events preceding APs in pyramidal cells were first described and called fast prepotentials (Spencer and Kandel, 1961, Figure 1).

**FIGURE 5:**
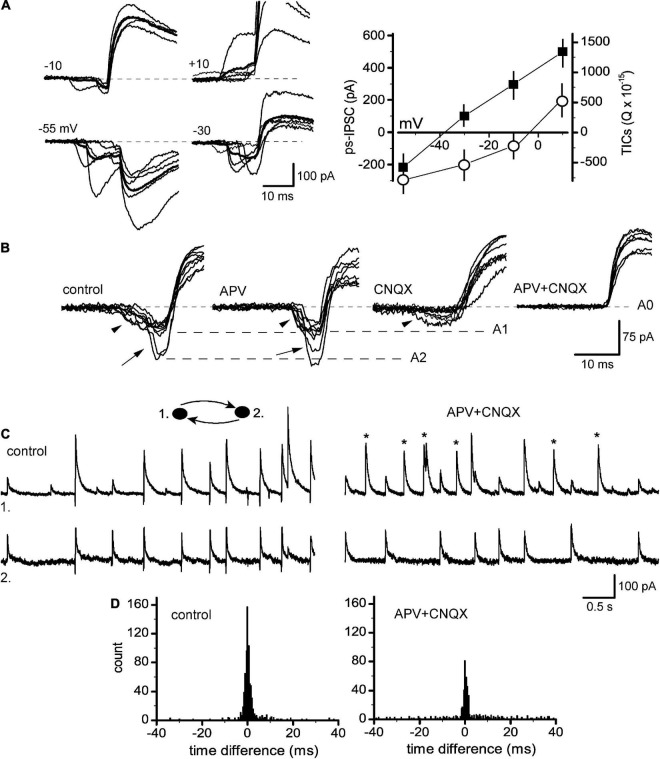
Glutamate-dependent post-stimulus sIPSCs. **(A)** TICs/ps-IPSCs sequences at different membrane potentials (*left*), lines in *thick* show averages (*n* = 10). *Right*, respective *I-V* curves plotted for both TICs (as integrals, *open circles*) and ps-IPSCs (peak amplitudes, *filled squares*). **(B)** Expanded and superimposed segments showing variety in TICs waveforms consisting of, respectively slow and small components (*arrowhead*) and fast transients (*arrow*), those fluctuated in an all-or-none way between levels *A1* and *A2*. Traces are shown in control, under APV, CNQX and in both. **(C)** Recording of ps-IPSCs in control (*left*) and under APV + CNQX (*right*), TICs were abolished and uncorrelated sIPSCs appeared (*stars*). **(D)** Respective time difference histograms.

The data above indicated that TICs, despite their heterogenous shape, were excitatory and synchronizing events reminiscent of sEPSCs, which prompted the question of whether they represented the same or distinct entities. To resolve this issue, ion dependency and pharmacological sensitivity of TICs were estimated. Spontaneous ps-IPSCs were recorded at different potentials and *I-V* curves were plotted for both excitatory and inhibitory parts (Figure 5A). In the majority but not all of the cells (*n* = 12/17), TICs behaved similarly to sEPSCs reversing their direction at −4.0 ± 1.3 mV (*n* = 6), which suggested permeation of both Na^+^ and K^+^. Interestingly, the reversal potential of the inhibitory part (−39 ± 2.5 mV, *n* = 6) was less negative than that of eIPSCs, presumably due to contamination of their rising phase with excitatory currents. The role of iGluR was tested next by applying APV and CNQX, selective antagonists of NMDA and AMPA/kainate receptor subtypes (Figure 5B). The drugs were used alone or in combination with the purpose of identifying distinct kinetic components of TICs (arrowhead and arrow). Slowly rising currents (arrowheads) with an amplitude up to A1 were modified by APV and CNQX becoming faster and slower, respectively, indicating that they were mediated by both subtypes of iGluR. In contrast, faster and larger transients (arrows) behaved differently and apart from being abolished by CNQX, they revealed properties of voltage-activated channels. Namely, those events occurred in an all-or-none mode fluctuating between A1 and A2 at V_*m*_ −30 mV and suddenly disappeared with further small depolarization, at −21 mV in a given case (not shown), apparently due to channels inactivation. These features suggest that these fast transients were active electrical events (spikelets) generated locally in remote sites of a given cell. Eventually, the combined application APV/CNQX abolished all inward currents (Figure 5B, right). The blockage of TICs was accompanied by reduced synchrony of ps-IPSCs and the appearance of uncorrelated sIPSCs (Figure 5C). In the time difference histogram it was evident as a lower central peak and decreased *R*-value, 43.5 ± 2.8% vs. 79.4 ± 3.1 (*n* = 12, *t* = 11.2, *P* < 0.001; Figure 5D).

**FIGURE 6:**
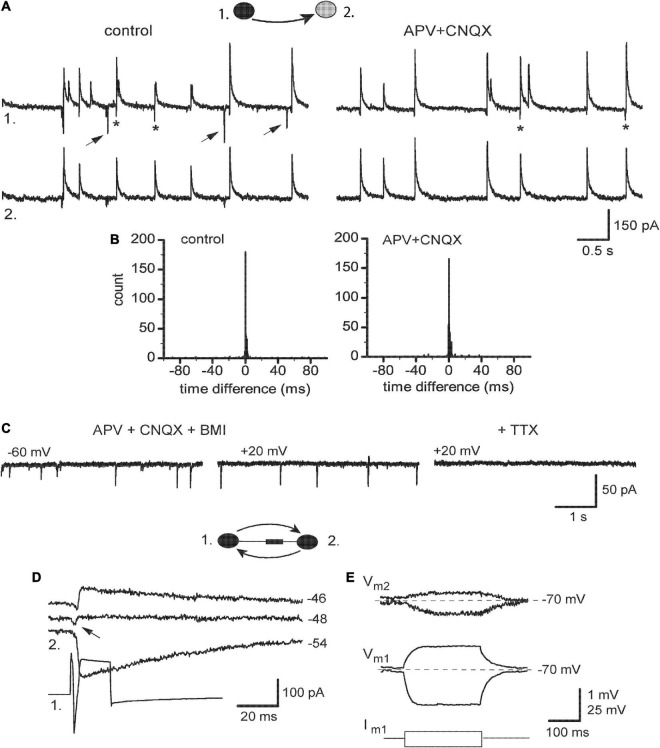
Expression of electrical coupling in ps-IPSCs weakly dependent on iGluR. **(A)** Pair recording of ps-IPSCs in control and under 100 μM APV with 20 μM CNQX. Some of the random sEPSCs are marked by *arrows*, and TICs–by *stars*. **(B)** Respective time difference histograms. **(C)** Spontaneous fast transients occurring under blockers of iGlu and GABA_A_ receptors and insensitive to membrane potential, which were then abolished by 0.5 μM TTX. **(D)** Combined electrotonic (*arrow*) and GABAergic responses induced by presynaptic stimulation (*1*) in postsynaptic cell (*2*) in voltage-clamp mode. **(E)** Current-clamp recordings, depo- and hyperpolarizing voltage responses induced in cell 1 (V_m1_) were passively transmitted to cell 2 (V_m1_).

The remaining five pairs revealed partial sensitivity of their TICs to blockers of iGluR even in higher concentrations, while sEPSCs were fully abolished (Figure 6A). This was paralleled by their smaller effects on the synchrony of ps-IPSCs. In time difference histograms, the height of peaks was almost unchanged and the *R*-value decreased slightly, from 83.6 ± 5.1 to 73.2 ± 3.1% (*n* = 5, *t* = 2.17, *P* > 0.05; Figure 6B). To identify the nature of these iGluR-independent components of TICs, they were isolated pharmacologically in a mixture of APV, CNQX, and BMI appearing as fast inward transients at −60 mV (Figure 6C, left). Strong membrane depolarization to +20 or +40 mV was unable to affect them (middle) but the further application of 0.5 μM TTX abolished all currents (right). This insensitivity to voltage and synaptic blockers suggested that remaining TICs were passive electrotonic currents reflecting APs in nearby neurons and transmitted presumably *via* gap junctions. In support, presynaptic stimulation revealed combined chemical and electrical (arrow) responses in 3/5 pairs belonging to the IN-IN type (Figure 6D). Remarkably, no electrical coupling was observed in mixed IN-EN pairs. Passive subthreshold responses were also transmitted between connected neurons kept in current-clamp mode and the coupling ratio for DC signals was ∼1.5% (Figure 6E).

**FIGURE 7:**
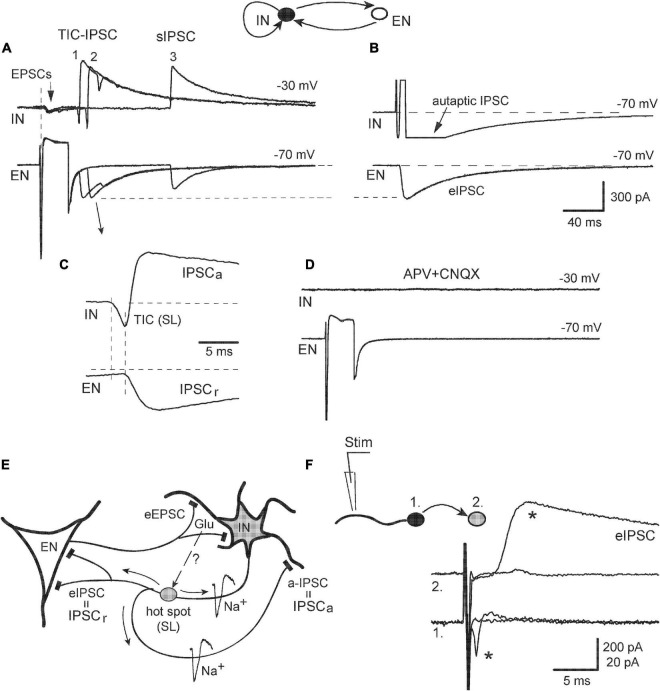
Example of the electrical behavior of excitatory-inhibitory cell circuit. **(A)** Presynaptic stimulation of EN induced three kinds of postsynaptic responses (three of them were superimposed): monosynaptic EPSCs (*arrow*), remote sequences TIC-IPSC (*1*, *2*), and late sIPSC (*3*). **(B)** Stimulation of IN evoked IPSC in EN and autaptic IPSC in itself (it was blocked by BMI). **(C)** TIC-IPSC sequence #2 was expanded to show its intrinsic temporal relations (see Text for details). **(D)** The application of APV + CNQX blocked all responses. **(E)** Schema describing electrical behavior of EN-IN pair. TICs appeared as local spikelets (*SL*); they originated in axonal *hot spots* and induced both autaptic IPSC (*a-IPSC*) and *eIPSC* in EN. Putative signaling of synaptic Glu to axonal iGluR is shown by a *dashed arrow* and *question mark*. **(F)** A test of the active electrical role of the dendrites. Local extracellular stimulation of a dendrite in the IN (*1*) induced all-or-none APs (*star*) in the soma and corresponding eIPSC (*star*) in the follower cell (*2*).

The dominant role of iGluR in the initiation and mediation of synchronized ps-IPSCs suggested a primary and causative contribution of excitatory neurons releasing Glu synaptically. This aspect was investigated in recordings from IN-EN pairs and revealed complex cell interactions beyond simple monosynaptic responses (Figure 7). Stimulation of pyramidal-like EN evoked in IN not only typical EPSCs with a short delay and no failures (A, arrow) but also, in ∼40% of pairs (*n* = 4/9), two kinds of remote synchronous responses: TIC-IPSC sequences with a delay of 40–50 ms and ∼20% of failures (*1*, *2*) and late sIPSCs appearing after 100–200 ms with ∼50% of failures (*3*). Late sIPSCs had no pre-currents and were likely due to the firing of a third cell, in accordance with data in Figure 2. Most relevant and remarkable was the finding that the activity of EN in a simple circuit could generate TIC-IPSC sequences mimicking network-induced ps-IPSCs. The analysis below demonstrates one of the ways how population events could be organized. Sequence #2 is expanded on in Figure 7C and shows temporal relations between its components: TIC occurring first and then paired IPSCs (IPSC_*a*_ and IPSC_*r*_). The latency between them, if measured from TIC onset (its peak was contaminated by IPSC_*a*_) and thus overestimated, reached 2.0 ± 0.1 ms (*n* = 4, *P* < 0.01) being much smaller than the synaptic delay of eIPSCs (∼2.7 ms). Induced TICs consisted of only fast transients occurring in an all-or-none mode and similar to spontaneous ones (Figure 5B, arrows), were classified as spikelets (SLs) generated locally in remote sites. Similarly, these SLs originated in a given IN because cell inactivation by membrane depolarization to −18 mV abolished SLs along with the simultaneous disappearance of paired IPSCs. In turn, the latter suggested that SLs were causal in initiating both IPSC_*a*_ and IPSC_*r*_, which specified their site of origin in the axonal tree of IN as intermediate between soma and synapses (designated as *hot spot* in Figure 7E). The axonal location of LSs helped to clarify next the origin of associated IPSCs. The IPSC_*r*_ in EN was a usual recurrent IPSC in response to a spike in IN, either generated locally in a *hot spot* or induced by somatic stimulation (confirmed by the same amplitudes of eIPSC in panel B and IPSC_*r*_ in panel A, *dashed line*). In addition to typical eIPSC, IN stimulation also induced an autaptic IPSC seen as a long tail after a depolarizing pulse (B); the IPSC_*a*_ then could be explained as autaptic IPSC in response to SLs propagating back from the *hot spot* to the soma (Figure 7E, a-IPSC). These effects of EN stimulation were almost fully blocked by APV/CNQX confirming the dominant, but not exclusive, role of iGluR in network activation (Figure 7D). Some late sIPSCs (as in Figure 7A, #3) in a few recordings persisted under the blockers suggesting additional involvement of metabotropic GluR (Miles and Poncer, 1993).

Identification of the axonal origin of some TICs was at odds with one of the current views ascribing the origin of spikelets to the dendritic tree, as studied in pyramidal cells (Spencer and Kandel, 1961). In order to get more information on whether the dendrites of INs in our model could play an active role in electrogenesis, we stimulated the neurites of a presynaptic IN (Figure 7F, cell #1, kept in attached mode) locally with an extracellular pipette and simultaneously observed postsynaptic neuron (#2) in whole-cell mode. Really, active dendritic sites were found at distance of 50–70 μm from the soma stimulation which evoked, first, back-propagating APs and then corresponding eIPSC (stars). Thus, the dendrites of hippocampal INs *in vitro* were also capable to generate locally active electrical events and be the site of TICs origin.

## 4. Discussion

Mechanisms of interneuronal synchronization were studied in a simple model network during a dynamic transition from the rest to moderate activation with intact synaptic transmission, which presumably mimicked physiological processing *in vivo*. Resting INs fired individually and synchrony of sIPSCs did not require a special mechanism. We report that network activation resulted in the coherent firing of a few INs recruited by excitatory synchronizing currents (TICs). This was evident in the appearance of “hypersynchronous” population events, compound sIPSCs temporally preceded by TICs. The network nature of TICs was evident by their heterogeneous components, Glu-mediated currents, local dendritic and axonal spikelets, and coupling currents transmitted likely *via* gap junctions. We found then that network-driven population events could be initiated by synaptically released Glu and generated within a minimal circuit consisting of reciprocally connected excitatory and inhibitory neurons.

### 4.1. Divergence of inhibitory axons

Even in the resting network, a large part of sIPSCs occurred synchronously, which was abolished by TTX showing the necessary role of APs in any kind of correlated neural activity (Ben-Ari et al., 1989; Vincent and Marty, 1993; Fischer et al., 2002). In their turn, the quantal release of neurotransmitters from individual release sites is stochastic and asynchronous between cells (Ropert et al., 1990). The properties of APs-matched sIPSCs and unitary eIPSCs were similar (Figures 2F, G), and the firing of single INs could evoke IPSCs in several postsynaptic cells (Figure 2D). This suggested strongly that sIPSCs were unitary events and their correlation resulted from APs arising in a single presynaptic IN (see also, Miles, 1990b; Vincent and Marty, 1993). The latter can be achieved due to the divergence of axon collaterals of hippocampal interneurons, which are known to target many pyramidal cells and INs (Buhl et al., 1994; Gulyás et al., 1996). Obviously, this type of synchrony in sIPSCs is passive and does not require a special mechanism. Interestingly, the spontaneous firing of resting INs seemed to be driven endogenously without clear excitatory input because presynaptic APs arose from a flat baseline without preceding TICs (Figures 2E, F). Presynaptic APs propagated then a small distance (<150 μm) toward release sites and small fluctuations at this stage explain minimal time differences between paired sIPSCs (<1 ms).

### 4.2. Excitatory synchronization of interneurons

After field electrical stimulation was over, the neural behavior changed qualitatively resulting in the appearance of post-stimulus sIPSCs, the elevated synchrony of which clearly required some special mechanisms. Using currents separation by their driving force, initial excitatory components were resolved in seemingly homogenous inhibitory events. Thus, ps-IPSCs represented excitatory-inhibitory sequences (TICs/ps-IPSCs) occurring spontaneously. They were reminiscent of evoked excitatory-inhibitory responses to afferent stimulation in hippocampal slices (Alger and Nicoll, 1982; Miles and Wong, 1984; Davies and Collingridge, 1989; Lacaille, 1991). Further analysis revealed an intrinsic structure of post-stimulus sIPSCs, both parts of which were identified as population events: TICs consisting of several heterogeneous components and ps-IPSCs composed of 2–3 unitary sIPSCs. This temporal order with prior TICs and their ability to trigger APs unequivocally demonstrated that the former represented an exogenous excitatory drive to INs and a cause of increased coherence. The time width of excitatory synchronization was much broader (±4 ms) than that of baseline sIPSCs and included other processes apart from APs propagation. It was apparently determined by the kinetics of TICs and included at least one synaptic delay (e.g., Figure 4B left). Another peculiar feature of ps-IPSCs was displaced time difference histograms in ∼50% of IN-EN pairs (see also Vincent and Marty, 1996). We hypothesized that it was due to asymmetry in local cellular interactions during the build-up of population events, e.g., a cell with earlier and/or larger TICs in a pair could play a more active role in local boosting of synaptic inhibition. This idea was supported in the analysis of synaptic responses in EN-IN pairs (Figure 7A). Stimulation-induced TICs occurred first in an axonal tree of INs and initiated both recurrent and autaptic components of paired IPSCs (Figures 7C, E). The active and necessary role of INs was confirmed by their voltage inactivation or even destruction by a gentle pressure of the recording pipette, which abolished TIC-IPSC sequences.

Remarkably, the firing of a single EN within a minimal synaptic circuit with one IN was necessary and sufficient to induce excitatory-inhibitory sequences (Figure 7A), which largely mimics field electrical stimulation. This indicated a principal source of excitation for INs and highlighted a primary role of synaptically derived Glu. According to our data, the occurrence of such sequences either spontaneously in culture or evoked by afferent stimulation in slices is to be expected because they are a signature of the activity in simple but ubiquitous reciprocal circuits consisting of only ENs and INs. Relevantly, stimulation of single pyramidal cells in hippocampal slices under the presence of GABA_*A*_ blockers also led to network activation and to the development of epileptiform bursting activity (Miles and Wong, 1983). Thus, the physiological sense and necessity of EN-IN circuits and of its electrical epiphenomena seem clear, to restrict neural over-excitation and promote the processing of afferent signals. While the neocortex is a brain region developmentally close to the hippocampus, recordings from EN–IN pairs therein showed simple unitary EPSPs but no signs of network activation (i.e., excitatory-inhibitory sequences) were evident (Karnani et al., 2016); the latter could be due to differences in animal species, local synaptic connectivity, etc.

Single-cell and field electrical stimulations had also two distinctions. First, TICs occurred in only one cell of a pair, which was likely due to a singular source of excitation, while numerous cells were excited by field stimulation. Second, TICs consisted of only fast transients seemingly without prior and slower Glu components (compare Figure 5B vs. Figure 7C). The reasons for this remain unclear, especially because Glu was surely released upon stimulation and evoked EPSCs *via* activation of somato-dendritic iGluR. One possibility was that Glu also diffused a long distance toward axonal iGluR concentrated within a hot spot (Figure 7E, dashed arrow). The existence of both axonal and preterminal iGluR of the kainate subtype was suggested for hippocampal INs in slices (Cossart et al., 2001; Semyanov and Kullmann, 2001). Relatively slow Glu potentials would be then filtered out due to their remote electrical location and poorly detected in the soma. Overall, while the deduced schema describes the behavior of EN-IN pairs *in vitro* (Figure 7E), it might be not fully applicable *in situ*. More specifically, autaptic IPSCs seem to be over-expressed in culture (Mennerick et al., 1995), while their existence in slices has been only suggested but not yet proven (Miles, 1990a; Gulyás et al., 1993).

Despite the principal similarity between spontaneous excitatory-inhibitory sequences in culture and evoked afferent responses in slices, there is, however, a major distinction in their first components. The excitatory phase of afferent responses was blocked by APV/CNQX indicating that it was a simple evoked EPSP mediated solely by iGluR (Davies and Collingridge, 1989; Lacaille, 1991). In contrast, spontaneous TICs were composed of several heterogeneous components and were not limited to iGluR. This distinction is to be expected though because both electrical phenomena reflected different phases of network activity. Afferent responses induced by electrical or sensory stimulation only initiate network activity, which occurs *via* afferent fibers purely Glu-ergic in their nature. On the other hand, excitatory-inhibitory sequences occur spontaneously after stimulation is over. They show the intrinsic way of self-sustaining operation of the whole network, with its all available excitatory components. While those have been already described individually in the literature, our results suggest, however, that these means operate jointly during natural afferent processing. The network excitatory components driving the activity of INs are discussed below.

#### 4.2.1. Ionotropic GluR

The excitatory and synchronizing drive to pairs of neurons was mainly provided by iGluR (the role of mGluR was not yet studied), which agrees with classical knowledge on excitation of individual hippocampal INs (Miles, 1990a; McBain and Dingledine, 1993; Miles and Poncer, 1993; Geiger et al., 1997; Frerking et al., 1998; Semyanov and Kullmann, 2001). All cells had TICs with Glu-mediated currents, either independent or along with other components. When in combination with local spikelets, Glu current preceded and initiated them (e.g., Figure 5B). In the results, synchrony in ∼2/3 of pairs was fully dependent on iGluR. The remaining cells displayed coupling currents presumably *via* gap junctions and were weakly sensitive to iGluR blockers. The reasons for the latter remain unclear; possibly other agents apart from Glu were released in the activated network and promoted the expression of gap junctions (Fischer, 2004). In terms of receptor profile, all known subtypes of iGluR participated in TICs under the presence of extracellular Mg^2+^, i.e., AMPA/kainate and NMDA receptors. Those were activated either simultaneously (Figure 5B) or even individually given a large variety of TICs waveforms (see also, McBain and Dingledine, 1993). In turn, the synchrony of Glu-ergic input to INs (Figure 3E) was presumably mediated by divergence in axonal collaterals of excitatory cells (Miles and Wong, 1986; Miles, 1990a).

Paired recordings performed in neocortical INs have suggested the independence of their synchrony on iGluR (Tamás et al., 2000; Hu et al., 2011). Those studies, however, were focused on a distinct phenomenon, a near-synchronous correlation of APs firing observed in special conditions when both neurons in a pair were tonically/phasically depolarized *via* intracellular pipettes. It is clear, however, that *in situ* conditions such depolarization can only be produced by afferent presynaptic fibers, which necessarily release Glu on their targets.

#### 4.2.2. Local spikelets and fast prepotentials

The majority of fast components of TICs were active electrical events (here termed as spikelets) generated locally in remote sites of recorded INs because they were affected by manipulations on given cells. Such small, transient, and all-or-none depolarizations have been known for a long time as partial spikes, spikelets, or short latency depolarizations and, as they often preceded full APs, they were widely referred to as fast prepotentials (FPPs) (Spencer and Kandel, 1961; MacVicar and Dudek, 1981; Núñez et al., 1990; Michelson and Wong, 1994). In our recordings, spikelets were analogous with classical FPPs in preceding full APs (Figure 4E). Two current hypotheses explained FPPs either as active events occurring in dendritic sites of impaled neurons (Spencer and Kandel, 1961; Hu et al., 1992; Steriade et al., 1993) or coupling potentials in response to the activity of nearby neurons and propagated passively to recorded cells *via* gap junctions (MacVicar and Dudek, 1981). Both scenarios were found valid here (the latter will be discussed below). Active spikelets, in turn, were often preceded and initiated by slower Glu-mediated currents (Figure 5B) and therefore were apparently generated in the dendrites of INs, where iGluR are located. The active role of dendrites in electrogenesis has been demonstrated for pyramidal cells, which possessed voltage-activated channels and could generate APs in response to synaptic input (Regehr et al., 1993). Similarly, the dendrites of INs were also capable to generate electrical events at some spots upon local electric stimulation, which induced back-propagating APs in the soma (Figure 7F; see also, Martina et al., 2000). We added complexity to this picture further and revealed that some spikelets could be initiated locally in the axonal tree of INs, as they were capable to induce postsynaptic responses (Figures 7C, E). Ectopic initiation of APs in axonal terminals rather than in the axon initial segment was also observed in pyramidal cells and attributed to terminal hyperexcitability induced by tetanic stimulation (Stasheff et al., 1993). Such active axonal sites could be designated as “hot spots,” because they should contain an amount of iGluR to be responsive for synaptic Glu, in addition to voltage-activated Na^+^ channels (Figure 7E). The physiological sense of local axonal spiking in INs seems clear, to restrain neural over-excitation and boost synaptic inhibition.

#### 4.2.3. Gap junctional communication

A significant fraction of pure INs pairs (3/5) was weakly coupled electrically and displayed fast TICs due to the activity of nearby neurons transmitted passively. This did not occur in control conditions but was observed after network activation and mediated presumably *via* gap junctions (GJs). The lack of sufficiently selective pharmacological tools prevented us from more precise identification (Rouach et al., 2003). GJs allow permeation of electrical currents and small molecules and have been frequently observed along with FPPs in hippocampal neurons (MacVicar and Dudek, 1981; Michelson and Wong, 1994; Strata et al., 1997). Solid morphological support for GJs was obtained in rats and respective specializations were demonstrated at the ultrastructural level *in situ* (Gulyás et al., 1996; Fukuda and Kosaka, 2000). However, the functional consequences of GJs in slices are somewhat controversial depending on animal species and the plane of the section. In the latter case, they could be overestimated due to stronger experimental damage and subsequent membrane fusion in coronal slicing (Gutnick et al., 1985). No coupling was observed in guinea-pig hippocampus under normal conditions, but it appeared in epileptogenic tissue (Miles, 1990b; Gulyás et al., 1993; Michelson and Wong, 1994; Zhang et al., 1998). In contrast, a high fraction of INs (50–80%) was electrically coupled in rat cortex with a DC ratio of up to 10% and up to 20% in mouse hippocampus (Gibson et al., 1999; Beierlein et al., 2000; Tamás et al., 2000; Bartos et al., 2002). Cultured neurons after 2 weeks have already repaired their experimental damage due to isolation and are advantageous in this respect. Our recordings thus agree with some of the reported studies in slices and suggest the functional expression of GJs in activated hippocampal networks. Remarkably, neural activation could be achieved non-specifically by tetanic electric stimulation or blockers of K^+^ channels and once activated GJs did not require iGluR further (Michelson and Wong, 1994; Zhang et al., 1998; Skinner et al., 1999). Here too, coupling transients were induced and observed in combination with Glu currents much in the same way as active spikelets (Figure 5B) but they could persist thereafter even in the absence of Glu transmission (Figures 6A, B). Remains unclear, however, which mechanisms and neuroactive agents regulate the expression of GJs; in particular, the role of cholinergic agonists was proposed (Fischer, 2004). Clearly, further studies on this issue are warranted.

#### 4.2.4. GABAergic depolarization

As soon as we used a novel model of synchronized activity, we also tested the relevance of a special mechanism thought to be possible in synaptic connections of INs, namely the depolarizing and excitatory action of released GABA. The latter is feasible with sufficiently elevated intracellular Cl^–^ level rendering reversal potential for GABA (E_*GABA*_) positive to APs threshold. Such mechanism participated in network excitation and synchronous GDPs in the neonatal hippocampus but disappeared later in development after two postnatal weeks (Ben-Ari et al., 1989; Khazipov et al., 1997; Khalilov et al., 1999; Wester and McBain, 2016). On the other hand, depolarizing GABA responses are not a unique feature of immature neurons and could be also observed in adults, e.g., in some subsets of INs in baseline conditions (Szabadics et al., 2006; Fu and van den Pol, 2007) or even more importantly, during epileptiform activity induced chemically or by tetanic electrical stimulation shifting E_*GABA*_ to more positive values (e.g., Michelson and Wong, 1994; Staley et al., 1995; Benardo, 1997). Similarly, field electrical stimulation in our experiments (e.g., Figures 3A, B) might have increased postsynaptic Cl^–^ level and uncovered the excitatory action of GABA. Exploring such a hypothesis, however, is not a trivial technical task. It requires the following conditions: undisturbed intracellular ions in the postsynaptic neuron, its resting membrane potential, and adequate presynaptic stimulation. The first two criteria are fulfilled when keeping postsynaptic cells in attached configuration, while a third one has been routinely approximated so far by using extracellular electrical stimulation combined with the application of blockers to isolated GABA or Glu postsynaptic action. Using such an approach, conflicting conclusions were reached in young hippocampal INs (Khazipov et al., 1997; Khalilov et al., 1999) versus those in the cortex (Kirmse et al., 2015). To refine further the protocol and avoid uncertainties of extracellular stimulation (discussed in Miles, 1991), we stimulated identified presynaptic INs individually, while recording postsynaptic neurons in attached mode. No APs were induced in postsynaptic cells, both INs and ENs (Figure 1B, trace 1), in contrast to presynaptic stimulation of ENs (Figure 1C, trace 1), which was the case both in control conditions and after moderate network stimulation. These results, however, are suggestive and cannot be fully applicable to *in situ* conditions, because they did not include modest depolarizing action of HCO_3_^–^ ions (Staley et al., 1995). Moreover, it is not excluded that stronger stimulation would increase GABA-mediated depolarization up to postsynaptic excitation.

### 4.4. Population IPSCs

Here we described population sIPSCs (in the text as ps-IPSCs) and identified them as a distinct type of inhibitory events arising due to the coherent firing of a few presynaptic INs. They seem to represent a necessary feature in the organization of synaptic inhibition and need to be compared with prior studies using slices. It should possibly come with no surprise that population events were not previously recognized in descriptions of sIPSPs or sIPSCs in resting neurons and under blockers of iGluR (Miles, 1990b; Otis and Mody, 1992; Hájos and Mody, 1997; Williams et al., 1998). On the other hand, the existence of population IPSPs has been tentatively presumed in highly activated networks, which was based on dissimilarities of those events with single-cell induced unitary IPSPs (Miles and Wong, 1984, Figure 8A; Fischer et al., 2002, Figure 2). Other lines of evidence have relied on some temporal correlations (including negative delay) between APs in INs and sIPSPs occurring in partner cells in pair recordings, those pairs, however, were not synaptically connected and thus any perceived correlations could be stochastic (Benardo, 1997, Figure 5A; Velazquez and Carlen, 1999, Figure 3A; Beierlein et al., 2000, Figures 5A, B). Similar temporal analysis was used here but in synaptically connected neurons. This verified the causal relationship between APs and sIPSCs and thus showed unambiguously the compound nature of population sIPSCs as consisting of several unitary events (Figures 4B–D). The peculiar feature in our recordings, however, was the association of population sIPSCs with excitatory pre-currents, which has not been regularly observed before, except for network-driven GDPs (Khalilov et al., 1999, Figure 3C left; Wester and McBain, 2016, Figure 1Eiii). The likely explanations could be the usage of sharp microelectrodes having lower amplitude and frequency resolution, recording at negative V_m_ when excitatory and inhibitory currents were of the same direction, and the presence of iGluR blockers in some studies. Also, it cannot be fully excluded that excitatory-inhibitory sequences were an artifact of culture, however, the integrity of presented data strongly suggests that they correspond to the natural organization of interneuronal activity.

## Data availability statement

The original contributions presented in this study are included in the article/supplementary material, further inquiries can be directed to the corresponding author.

## Ethics statement

All animal procedures here conformed to the principles of world-wide regulations (Grundy, 2015). The experiments were carried out according to guidelines approved according to Protocol No. 3/14 from 06.2015 from the Bogomoletz Institute of Physiology (Ukraine) and as regulated by the European Community Council Directive (2010/63/EU).

## Author contributions

VP prepared hippocampal culture and participated in electrophysiological experiments and data analysis. IM designed this project, led the experiments and data analysis, and wrote the manuscript. Both authors contributed to the article and approved the submitted version.
